# The Cost of Accessibility: Considerations for Adopting Microscope Camera-Derived Images for Digital and Computational Pathology

**DOI:** 10.1016/j.mcpdig.2026.100379

**Published:** 2026-06-08

**Authors:** Jakob N. Kather, Matthew G. Hanna, Chris Garcia, Jill Stefanelli, Richard Levenson, John W. Longshore, Felix Faber, Ross J. Hill, Luiza Moore

**Affiliations:** a Else Kroener Fresenius Center for Digital Health and Department of Medicine I, University Hospital Carl Gustav Carus, TUD Dresden University of Technology, Germany; b Department of Pathology, University of Pittsburgh Medical Center, PA; Computational Pathology and AI Center of Excellence (CPACE), University of Pittsburgh, School of Medicine, PA; c Department of Pathology, Mayo Clinic, Rochester, MN; d Modella AI, BO; e Department of Pathology and Laboratory Medicine, University of California Davis, CA; f AstraZeneca, Durham, NC; g Mindpeak, Hamburg, Germany; h AstraZeneca, Cambridge, UK

The digital transformation of pathology has accelerated over the past decade,^1^, ^2^, ^3^, ^4^, ^5^ driven by advances in whole-slide imaging (WSI) and artificial intelligence (AI)-enabled solutions for biomarker assessment.^6^, ^7^, ^8^ WSI represents the gold standard for digital pathology (DP), providing comprehensive tissue digitization with established and validated regulatory frameworks. However, capital costs and substantial infrastructure requirements currently limit broad global adoption,^9^ with approximately 20% of pathology laboratories having access to whole-slide scanners and as few as 5% of laboratories employing digital workflows for routine diagnostic reporting.^10^^,^^11^

Microscope-camera imaging (MCI)—the acquisition of digital images using cameras mounted on conventional brightfield microscopes—represents a pragmatic, cost-effective, and accessible route to DP images for biomarker assessment.^2^^,^^12^ MCI leverages the ubiquitous microscope infrastructure already present in pathology laboratories worldwide, providing a viable route to digital workflows for resource-constrained laboratories unable to adopt WSI. Indeed, given that the origins of DP lie in photomicrographs captured by modest microscope cameras, it is only logical, and perhaps urgently necessary, to re-examine the role of microscope camera-derived images within contemporary digital and computational workflows.

However, the reliable adoption of MCI depends on overcoming substantial challenges in standardization, validation, and regulatory acceptance. Unlike WSI, MCI workflows currently lack clear guidelines, and the heterogeneity of microscope-camera hardware combinations introduces variability in image quality that must be systematically addressed. For MCI to serve as a viable pathway to expand access to digital and computational pathology (CP), coordinated efforts across the pathology community, academia, industry, and regulatory bodies are essential to develop technical standards and validation frameworks. Here, we discuss the potential role of MCI, which, unlike WSI, represents an abundant yet underutilized digital resource in the context of clinical diagnostic workflows, particularly for brightfield immunohistochemistry biomarker assessment (Figure).

**Figure:**
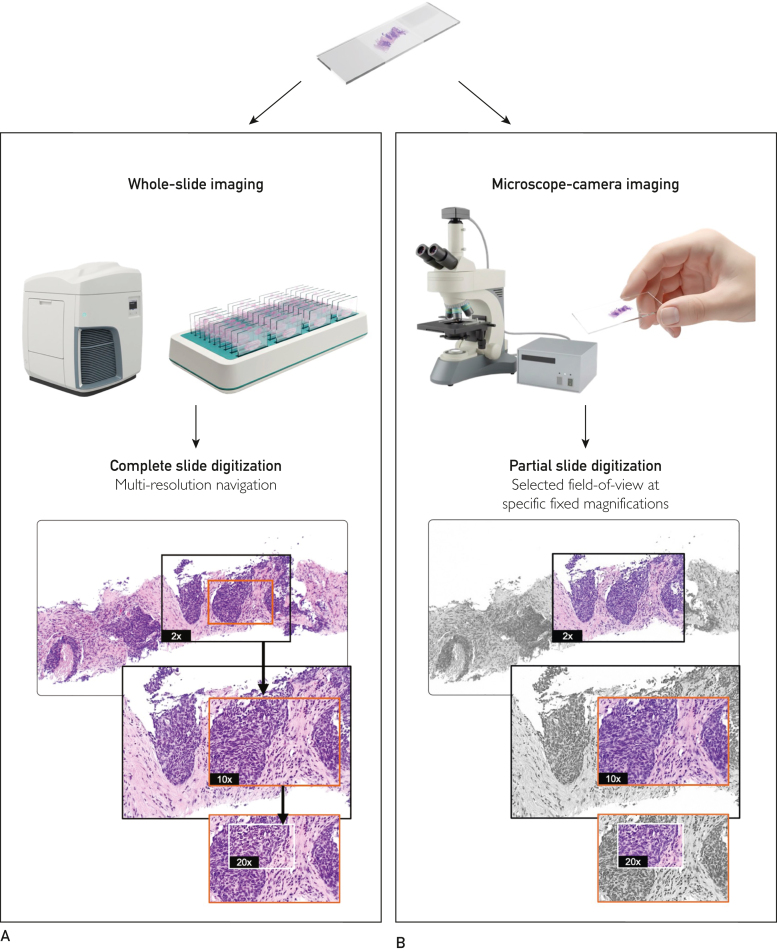
Comparison of whole-slide imaging (WSI) and microscope-camera imaging (MCI) hardware, workflows, and diagnostic interfaces. (A) WSI provides comprehensive, automated slide digitization that enables pathologists or other laboratory personnel to comprehensively review histologic specimens via multi-resolution navigation of whole-slide images. WSI workflows facilitate high-throughput sample digitization via the simultaneous loading of numerous slides within a scanning rack that are sequentially digitized through automated slide loading, tissue detection and image acquisition. The diagnostic interface of WSIs includes sample thumbnail overview, seamless image panning, and magnification zoom control across the entire tissue section. (B) MCI utilizes cameras mounted on standard brightfield microscopes and requires the manual positioning of a glass slide on the microscope stage, manual field-of-view (FOV) selection and image capture to produce individual, high-quality static images. The manual nature of MCI workflows may introduce additional operator time requirements compared to automated WSI workflows. The diagnostic interface of MCI-derived images typically includes a single static image, often with limited spatial context, highlighting the potential for sampling bias in FOV selection. Image attribution: The image depicts a H&E-stained non-small cell lung cancer specimen originally created by Librepath (Wikimedia Commons), licensed under CC BY-SA 3.0 (https://creativecommons.org/licenses/by-sa/3.0/). The image has been cropped and incorporated into this figure with panel B containing a modified image.

### Unlocking Access in Resource-Constrained Settings

In comparison to whole-slide scanners, microscope cameras are relatively low-cost and can be integrated into clinical workflows with minimal disruption. MCI systems produce high-quality single field-of-view (FOV) images with substantially smaller storage requirements. Indeed, WSI file sizes generally range from 0.3-4.0 gigabytes, depending on the size of the tissue section, scanning resolution, scanner model, and image compression.^4^^,^^13^, ^14^, ^15^ In contrast, microscope camera-derived image files are typically between 0.02-0.1 gigabytes, representing a 10-40-fold reduction in file size when compared with WSIs.^16^ This substantially lower storage burden reduces requirements for expensive infrastructure, high-speed network connectivity, and dedicated IT support staff, enabling laboratories with limited technical infrastructure to implement DP workflows without major investment. Additionally, microscope cameras can be purchased with, or retrofitted to, most brightfield microscopes, often without requiring infrastructural changes, and have long been adopted by the pathology community for educational activities, multidisciplinary team meetings and telepathology.^17^^,^^18^

However, although MCI reduces capital and infrastructure costs compared to WSI, it does not eliminate all economic barriers to CP adoption. Limited reimbursement for AI-enabled image analysis represents a significant ongoing challenge for both WSI- and MCI-based CP workflows. In the United States, existing CPT codes for computer-assisted image analysis often inadequately cover operational costs, such as software licensing and personnel time. This reimbursement gap affects CP adoption regardless of imaging modality.

Nevertheless, microscope-camera-derived images are already adopted in selected practices for biomarker quantification and secondary consultation.^19^, ^20^, ^21^, ^22^ Perhaps the most promising and immediate use-case for MCI is to facilitate the integration of AI-enabled algorithmic assessment of specific immunohistochemistry biomarkers (eg, human epidermal growth factor receptor 2 [HER2], estrogen receptor [ER], progesterone receptor [PR], and Ki67 in breast cancer). These applications could be particularly transformative in regions and institutions where access to WSI is not feasible. However, realizing this potential requires addressing substantial technical, operational, and regulatory challenges, which are discussed below.

### Technical and Operational Challenges

The adoption of MCI to facilitate digital and computational workflows raises several technical considerations compared with WSI, as summarized in Table. Most critically, heterogeneity in microscope-camera combinations and operator-dependent settings introduce substantial variability in image quality that may impact downstream visual or algorithmic analyses.^23^ Indeed, deep learning-based biomarker algorithms are sensitive to image quality differences, and the numerous hardware variables in MCI systems represent substantial standardization challenges. Operationally, however, integrated microscope cameras offer a minimally disruptive transition from traditional analog workflows to digital workflows.

**Table:** Summary of Key Considerations for Microscope-Camera Imaging (MCI) Implementation in Digital and Computational Pathology Workflows

Domain	Implementation consideration	Rationale
Hardware standardization and calibration	Microscope configuration; standardize objective magnification (eg, 40×), software settings; lock image acquisition parameters (eg, exposure time), hardware settings; standardize illumination intensity and condenser aperture (document any adjustments), reference standards; use certified stage micrometer for spatial calibration, color calibration slides (eg, Serra slide), control slides with known biomarker expression levels, MPP verification; Ensure ≤0.25 μm/pixel (40× equivalent), Metadata capture; document all imaging parameters	Heterogeneity across MCI hardware, operator adjustable software settings and environmental factors compounds image quality and variability
Field-of-view sampling	Homogeneous biomarkers (eg, ER, PR); 3-5 FOVs or number achieving >95% concordance with standard method, heterogeneous biomarkers (eg, HER2, PD-L1); 10-20 FOVs at 40× to capture spatial heterogeneity, Ki67 assessment; 3-5 FOVs capturing ≥1000 tumor cells, default approach; systemic sampling (eg, grid-based or random FOV selection), hot spot sampling; reserved for clinically validated applications, document rationale, documentation; record FOV selection strategy, coordinates, magnification, reproducibility; report consistency between operators	Individual or limited FOVs may fail to adequately capture intratumoral biomarker heterogeneity
Quality assurance	Daily QC; run control materials at multiple expression levels, longitudinal monitoring; assess QC trends over time, acceptance criteria; establish written workflow quality standards, change control; document and validate any MCI configuration changes (eg, hardware upgrades and software updates), performance monitoring; track concordance rates with reference method	Hardware variables, software changes and operator-dependent factors can impact image quality and downstream visual or algorithmic assessment
Personnel training	Qualification; high-complexity testing personnel, technical training; microscope setup, camera operation, focus optimization, quality assessment, histological training, tumor vs stroma distinction, invasive vs in situ distinction, artifact exclusion, competency assessment; eg, achieve >90% concordance with established method	Unlike WSI, MCI requires manual FOV selection, therefore personnel must recognize appropriate diagnostic areas for sampling
Workflow validation	Sample size; 20-40 samples per category (positive/negative), concordance; eg, ≥95% agreement with established method, Comparison method; FISH, consensus IHC scoring using established workflow, proficiency testing programs, configuration-specific; each microscope-camera combination may require independent validation	MCI encompasses heterogeneous microscope-camera combinations, each requiring independent validation

Reference standard sources: certified stage micrometers with 0.01mm graduations available from major microscopy vendors and laboratory distributor (eg, Labtech Agar Scientific, Aptex); color calibration slides such as the Sierra slide (PathQA Ltd); and control slides available from commercial vendors or through proficiency testing programs (eg, CAP surveys, UK NEQAS).

Abbreviations: ER; estrogen receptor, FISH; fluorescence in situ hybridization FOV; field-of-view, HER2; human epidermal growth factor receptor 2, MCI; microscope-camera imaging, MPP; micros per pixel, PD-L1; programmed death-ligand 1, PR; progesterone receptor, QC; quality control, WSI; whole-slide imaging.

### Hardware Heterogeneity and Calibration Requirements

The wide range of brightfield microscopes, each potentially paired with a variety of different camera models, creates significant heterogeneity in MCI systems, resulting in variability in image quality, resolution, and color fidelity. Integrated microscope cameras utilize the optical configurations of the adjoined microscope, including the objective lens magnification (eg, 4×, 10×, 20×, 40×, and potentially 63× or 100× oil-immersion lenses), condenser, camera adapter magnification (eg, 0.5×, 1.0×, and 2.5×), light source, and potentially the ocular lens depending on the camera integration method.

In stark contrast to whole-slide scanners with predetermined and locked settings, microscope-camera software frequently allows operator adjustments to image acquisition parameters, such as white balance, exposure time, gain, gamma correction, color contrast and color saturation. Beyond software adjustability, microscope hardware settings, such as illumination intensity and condenser aperture, can be easily altered between imaging sessions, introducing additional variability. Finally, MCI systems are potentially subjected to environmental factors, such as ambient light, that do not affect enclosed WSI systems.

Microscope cameras also vary in sensor quality, color balance, dynamic range, and image resolution (determined by pixel count in the imaging chip). Critically, differences in both camera resolution and microscope optical setup result in varying micron-per-pixel (MPP) values, meaning identical tissue structures may be represented at different spatial scales across independent images. MPP is calculated as:

$$MPP\;\left(um/pixel\right)=\frac{Sensor\;pixel\;size\;\left(um\right)}{Total\;magnification\;\left(objective\;x\;relay\right)}$$

Where the total system magnification is the objective lens magnification (eg, 20×) multiplied by any camera adapter (relay lens) magnification (eg, 0.5×), and the sensor pixel pitch is the physical size of a pixel on the camera sensor (typically 2.4-3.45 μm). For example, selecting either a 20× or 40× objective lens with a 0.5× relay magnification using a camera with a 2.4 μm sensor pixel pitch results in MPP values of 0.24 or 0.12 μm/pixel, respectively.

Therefore, acquiring images with consistent MPP values is essential to standardize downstream visual or algorithmic biomarker quantification. Coarse MPP (>0.7 μm/pixel) risks under-sampling biomarkers expressed in fine sub-cellular compartments such as nuclei or membranes, potentially causing quantification bias. Consequently, digital and computational pathology applications require ≤0.25 μm/pixel (40× equivalent resolution), with lower MPP values corresponding to increased resolution. Collectively, MCI system heterogeneity represents a relevant challenge for standardization of image quality, which may ultimately impact downstream digital and computational analyses.

#### Mitigation Strategies

To address these inconsistencies, rigorous standardization and calibration protocols are required, such as consensus guidelines on objective lens magnification selection (eg, 40×); standard operator procedures specifying acceptable ranges for imaging parameters with locked software settings where feasible; routine equipment calibration using validated reference standards (eg, certified stage micrometers for spatial calibration, validated color calibration slides such as the Sierra slide (PathQA Ltd),^24^ controls with known biomarker expression levels; quality control processes to ensure correct focal plane positioning and MPP verification; and regular operator training and competency assessment.

#### Personnel Requirements

Personnel operating MCI systems for clinical purposes must be appropriately trained and qualified; these may include pathologists, cytotechnologists, histotechnologists, or trained laboratory technicians depending on regulatory requirements, application complexity, and local institutional policies. Unlike comprehensive automated digitization by WSI, MCI necessitates manual image acquisition; therefore, training requirements must extend beyond technical operation to include histological feature recognition necessary for FOV selection, skills essential for ensuring diagnostically representative sampling. Finally, laboratories should follow strict documentation procedures to harmonize DP practices and capture sample metadata for traceability.^25^

### Mitigating Sampling Bias With FOV Selection

In stark contrast to whole-slide images, microscope-camera-derived images are typically single FOVs that are manually selected by the operator, introducing potential sampling bias. This poses significant limitations for many diagnostic tasks, including primary diagnosis and quantitative biomarker assessment. For example, a single or small number of FOVs may miss diagnostically valuable information, such as infrequent clusters of cancer cells with HER2 expression in a breast cancer specimen. Additionally, in the context of biomarker assessment, single FOVs may not appropriately capture complex intratumoral biomarker heterogeneity, potentially leading to misclassification (eg, the misclassification of a HER2 IHC 0 with membrane staining (HER2-ultralow) specimen as HER2 IHC 0 without membrane staining (HER2-null) if a single or small number of FOVs fails to capture the presence of partial, incomplete HER2 membrane staining present in ≤10% of tumor cells.

#### Standardization of Sampling Strategies

To minimize sampling bias, MCI procedural standards must evolve to incorporate robust mitigation strategies. The College of American Pathologists (CAP) guidelines for HER2 quantitative image analysis (QIA) emphasize the importance of well-documented procedures for region-of-interest (ROI) selection and require laboratories to report reproducibility between operators when selecting ROIs for analysis.^26^ Examples of sampling methods for HER2 QIA include selecting six ROIs at 40x magnification that represent the spectrum of HER2 expression within a specimen.^26^

#### Number of FOVs Required

The optimal number of FOVs depends on biomarker characteristics and spatial heterogeneity. For homogeneous biomarkers such as ER and PR in breast cancer, studies have demonstrated that 3-5 FOVs at 40× magnification can provide representative sampling, achieving 95% inter-reader concordance, comparable with manual whole-slide assessment.^27^ In contrast, heterogeneous biomarkers, such as HER2 and PD-L1, may require significantly more FOVs (eg, 10-20) to adequately capture spatial heterogeneity. For Ki67 proliferation assessment, the International Ki67 in breast cancer working group recommends scoring at least 1000 tumor cells,^28^^,^^29^ which would typically require 3-5 representative FOVs at 40× magnification. The sampling challenges inherent to MCI are analogous to tissue microarrays, where 2-3 systematically sampled cores can achieve >95% concordance with whole section assessment for biomarkers in breast cancer.^30^

#### Sampling Approaches

Systematic sampling strategies such as grid-based sampling or random FOV selection minimize selection bias compared to subjective identification of ROIs and should be the default approach for most biomarkers.^31^, ^32^, ^33^ However, for specific biomarkers where hot spot assessment is explicitly specified in testing guidelines (eg, Ki67 grading in neuroendocrine tumors),^34^^,^^35^ targeted FOV selection may be required.

#### Technological Solutions

Advances in image processing have enabled innovative approaches to address single FOV limitations, including the stitching of individual FOVs or the passive monitoring of pathologist assessment to generate WSI-like images,^36^ and augmented reality microscopes that directly overlay AI-enabled biomarker assessment projected through the eyepiece to aid pathologists in real-time assessment.^37^^,^^38^ These technologies may enable the seamless integration of CP solutions into diagnostic workflows without requiring major infrastructural changes.

#### Use-Case Insights and Limitations

The relevance and reliability of MCI-derived images are likely use-case dependent. Although the underlying technology is widely accessible, it is not universally suitable, requiring context-specific validation to establish diagnostic equivalence. MCI workflows cannot replace WSI for all diagnostic applications. Unlike WSI, where validation can be tied to specific scanner models with defined technical specifications, MCI encompasses a heterogeneous landscape of microscope-camera combinations, each potentially requiring independent validation.

Potential limitations are exemplified by HER2 assessment in breast cancer. Single or multiple FOVs may fail to capture intratumoral heterogeneity, risking biomarker misclassification. Additionally, lack of contextual information from single FOVs limits both visual and computational assessment, as ASCO/CAP guidelines state HER2 scoring should be carried out on the invasive component,^39^ requiring the exclusion of ductal carcinoma *in situ*, a distinction requiring contextual information to demonstrate confinement of abnormal cells by a basement membrane.

Nevertheless, MCI systems have been successfully implemented with pathologist-in-the-loop assessment of Ki67, ER, and PR (Mindpeak Breast Ki-67 RoI and Mindpeak ER/PR RoI) using QIA, achieving results concordant with manual pathology scoring and improved inter-reader agreement rates for HER2 assessment.^40^^,^^41^ Similarly, AI-assisted biomarker assessment using FOV imaging has demonstrated improved inter-reader concordance for PD-L1.^37^

#### Regulatory and Clinical Acceptance

Regulatory precedent for FOV-based diagnostics is well established. Food and Drug Administration (FDA)-cleared QIA systems for HER2 in breast cancer have utilized MCI-derived FOV images for over 2 decades.^26^^,^^42^ The 2019 CAP guidelines for QIA of HER2 establish validation requirements and performance standards applicable for both WSI and field-of-view approaches.^26^ Additionally, the ThinPrep imaging system (Cytyc Corporation) and the Panoptiq imaging system, which integrate microscope-camera-derived images with algorithmic prescreening for cervical cancer screening, have demonstrated the clinical utility and regulatory acceptability of FOV-based-imaging workflows.^43^^,^^44^

For MCI to be used in Clinical Laboratory Improvement Amendments (CLIA)-regulated environments, laboratories must establish that their specific MCI configuration produces images of equivalent diagnostic quality to the reference standard, whether that standard is conventional microscopy or WSI. This validation burden may be particularly challenging for AI-enabled tools, which are typically trained on WSI-only datasets and may require domain-specific retraining or extensive validation studies to be deployed on MCI-derived images.

However, contemporary regulatory questions remain regarding: (1) whether modern AI-enabled algorithms trained using WSI datasets require separate validation and clearance for MCI deployment; (2) how microscope-camera heterogeneity affect device performance and validation requirements compared to standardized FDA-cleared systems; (3) whether each laboratory's individual MCI setup constitutes a distinct Laboratory Developed Test (LDT) requiring independent validation under evolving LDT regulations; and (4) applicability of software as medical devices (SaMD) regulatory frameworks developed for WSI-based tools to MCI applications.

The development of consensus standards for MCI validation, including technical specifications, quality control metrics, and minimum acceptable performance criteria, will be essential for enabling broader adoption while maintaining diagnostic integrity.

Despite brightfield microscopes having long been categorized as Class I medical devices (510(k) exempt in the United States and Class I MDR Rule 1 in the European Union), the heterogeneity of MCI configurations and software-specific post-processing create uncertainty surrounding the regulatory path for contemporary MCI applications. In contrast, as of 2025, there are 7 whole-slide scanners with both FDA clearance (United States) and CE marking under the European in vitro diagnostics regulation (IVDR) for primary diagnostic workflows. Both WSI and MCI have established regulatory pathways for specific applications, though the landscape for novel AI-enabled tools continues to evolve, requiring further validation and potentially separate regulatory submissions for algorithm extension to MCI platforms.

### The Path Forward: Collaboration and Need for Structured Guidelines

Microscope-camera imaging stands at an inflection point. Although MCI has the potential to democratize access to DP and AI-enabled biomarker assessment, realizing this potential requires addressing substantial challenges.

Specific actions required include, as follows: (1) the development of consensus specifications for MCI hardware configurations suitable for clinical use, including minimum requirements for optical components and calibration protocols; (2) establishment of practical validation protocols tailored to heterogenous MCI systems, including alternative validation strategies for laboratories without access to WSI; (3) evidence-based recommendations for FOV sampling strategies that account for biomarker-specific spatial heterogeneity; (4) development of training curricula and competency assessment tools for laboratory personnel; and (5) clarification on regulatory pathways for AI algorithms deployed on MCI platforms, including domain adaptation requirements. Furthermore, technological solutions may further facilitate standardization, through AI-enabled quality control systems that automatically evaluate image quality (eg, illumination and color balance) and consistency over time.

Coordinated efforts across pathology societies, industry vendors, academic institutions and regulatory bodies are essential to develop these standards and guidelines. Such collaboration will enable MCI to serve as a viable pathway for expanding global access to CP while maintaining diagnostic quality and patient safety.

## Conclusion

Microscope-camera-derived images offer a pragmatic pathway to DP for resource-constrained laboratories by leveraging ubiquitous microscope infrastructure. However, hardware heterogeneity, operator-dependent variability, and FOV sampling challenges demand systematic standardization through the technical framework outlined in this commentary. Coordinated development of consensus standards for hardware calibration, validation protocols, quality assurance, and personnel training will enable MCI to democratize access to digital and computational pathology while maintaining diagnostic integrity.

## Potential Competing Interests

Dr Kather declares the following activities which are not related to the current work: ongoing consulting services for AstraZeneca, Panakeia, and Bioptimus. Furthermore, he holds shares in Stratifai, Synagen, and Spira Labs, has received an institutional research grant from GSK and AstraZeneca, and has received honoraria from AstraZeneca, Bayer, Daiichi Sankyo, Eisai, Janssen, Merck, MSD, BMS, Roche, Pfizer, and Fresenius. Dr Hanna is on an advisory board for PathPresenter, AstraZeneca, Johnson & Johnson, Roche, and Danaher Diagnostics. Dr Garcia has ongoing consulting services for Philips and has received honoraria from AstraZeneca and Roche. Dr Stefanelli holds equity in Modella AI. Dr Levenson is a cofounder of and holds equity in MUSE Microscopy, Inc. F Faber is a cofounder of and holds equity in Mindpeak. Drs Hill, Longshore, and Moore are employees of and hold equity in AstraZeneca.

## Declaration of Generative AI and AI-Assisted Technologies in the Writing Process

During the preparation of this work the authors used Claude in order to check readability and formatting. After using this tool, the authors reviewed and edited the content as needed and takes full responsibility for the content of the publication.
